# Histological and Radiological Assessment of Endogenously Generated Repair Tissue *In Vivo* Following a Chondral Harvest

**DOI:** 10.1177/19476035221149523

**Published:** 2023-01-26

**Authors:** Helen S. McCarthy, Bernhard Tins, Peter D. Gallacher, Paul Jermin, James B. Richardson, Jan Herman Kuiper, Sally Roberts

**Affiliations:** 1 Spinal Studies & Cartilage Research Group, Robert Jones and Agnes Hunt Orthopaedic Hospital NHS Trust, Oswestry, UK; 2 Centre for Regenerative Medicine Research, School of Pharmacy and Bioengineering, Keele University, Keele, UK

**Keywords:** articular cartilage, endogenous healing, trochlea, cartilage regeneration, osteoarthritis

## Abstract

**Objective:**

To examine repair tissue formed approximately 15 months after a chondral harvest in the human knee.

**Design:**

Sixteen individuals (12 males, 4 females, mean age 36 ± 9 years) underwent a chondral harvest in the trochlea as a pre-requisite for autologous chondrocyte implantation (ACI) treatment. The harvest site was assessed via MRI at 14.3 ± 3.2 months and arthroscopy at 15 ± 3.5 months (using the Oswestry Arthroscopy Score [O-AS] and the International Cartilage Repair Society Arthroscopy Score [ICRS-AS]). Core biopsies (1.8 mm diameter, *n* = 16) of repair tissue obtained at arthroscopy were assessed histologically (using the ICRS II and OsScore histology scores) and examined via immunohistochemistry for the presence of collagen types I and II.

**Results:**

The mean O-AS and ICRS-AS of the repaired harvest sites were 7.2 ± 3.2 and 10.1 ± 3.5, respectively, with 80.3% ± 26% repair fill depth on MRI. The histological quality of the repair tissue formed was variable, with some hyaline cartilage present in 50% of the biopsies; where this occurred, it was associated with a significantly higher ICRS-AS than those with no hyaline cartilage present (median 11 vs. 7.5, *P* = 0.049). Collagen types I and II were detected in 12/14 and 10/13 biopsies, respectively.

**Conclusions:**

We demonstrate good-quality structural repair tissue formed following cartilage harvest in ACI, suggesting this site can be useful to study endogenous cartilage repair in humans. The trochlea is less commonly affected by osteoarthritis; therefore, location may be critical for spontaneous repair. Understanding the mechanisms and factors influencing this could improve future treatments for cartilage defects.

## Introduction

Articular cartilage has long been thought incapable of healing itself once damaged, probably in part due to its lack of vascularization and limited nutrient supply.^1,2^ Furthermore, untreated cartilage defects tend to degrade further, commonly resulting in attrition of the articulating surface and eventually to osteoarthritis (OA).^3-6^ Autologous chondrocyte implantation (ACI) is a 2-stage cellular therapy, which is applied at an early phase in the potentially degenerative process. Culture-expanded chondrocytes isolated from macroscopically normal cartilage are harvested from a low load-bearing area of the patient’s joint (such as the trochlea) and are subsequently implanted into the cartilage defect beneath either a periosteal or collagen membrane,^7^ with good and sustained clinical outcome.^8,9^

Although degeneration of articular cartilage is generally thought to be irreversible,^10^ some studies have shown that untreated chondral defects, particularly deeper ones which expose or intrude into the underlying subchondral bone, can show some degree of natural repair in humans after 2 years.^5,6,11^ While it is possible to study the natural response and quality of repair tissue in a controlled manner to an injury in animals, this is not normally possible in humans.^12-16^ The harvesting procedure in ACI, however, potentially provides an opportunity to study the natural healing response following a standard and controlled injury in human articular cartilage. Little is known about the mechanisms that orchestrate cartilage repair in humans and, to date, there are limited human studies addressing or providing comprehensive insight into the mechanism of the self-repaired cartilage tissue. Pre-clinical large animal models to assess cartilage injury and repair mechanisms can provide some translational data, although biological differences such as anatomy, gait, and, therefore, loading, as well as cartilage thickness, require consideration.^17^

Previously, we have assessed the functional clinical outcome (Lysholm score) of a cohort of patients undergoing ACI in their hip or ankle, but with a chondral harvest from the (asymptomatic) knee and found no significant joint morbidity in the knee up to 4.8 years following this.^18^ Preliminary data in this cohort (*n* = 3) also demonstrated good arthroscopic and histological outcome.^18^ In the present study, we have investigated the quality of the endogenously repaired tissue formed following chondral harvest, using a combination of radiographical, histological, and immunohistochemical analyses.

## Methods

### Patients

Ethical approval was granted by the UK National Research Ethics Service (11/WM/0175), and written informed consent was received from all participants (*n* = 16). Each patient (12 males, 4 females, mean age = 36.9 years, range = 18-51, **Table 1**) received autologous cell therapy in our center for chondral/osteochondral defects in their knee. Chondral harvests were obtained using a 6-mm curved Capener gouge from the cranial femoral trochlea (mean weight of 278.2 ± 69.1 mg, range = 174-406 mg; **Table 1**) and recorded on a specially designed knee map.^19^ Harvests were processed in the on-site GMP, MHRA-licensed manufacturing facility (OsCell, John Charnley Laboratory, RJAH Orthopaedic Hospital, UK), according to established protocols.^20,21^ Chondrocytes were culture-expanded for approximately 3 weeks prior to implantation in the defect beneath a Chondrogide^®^ patch. All patients were offered an arthroscopy and biopsy of the harvest site at 12 to 15 months post-treatment, as part of their follow-up, according to the study protocol.

**Table 1.** Patient Demographics.

Patient Number	Age	Gender	Harvest Cartilage Weight (mg)
1	18	Female	197
2	21	Male	360
3	28	Male	296
4	29	Male	281
5	33	Male	329
6	35	Male	180
7	35	Female	312
8	36	Male	298
9	36	Female	192
10	37	Male	264
11	40	Male	325
12	42	Male	245
13	42	Female	174
14	47	Male	406
15	51	Male	n/a
16	51	Male	314

Each of the 16 patients included in this study are listed below with age, gender, and size of cartilage harvest taken. One patient’s harvest data (patient 15) was not available (n/a).

### MRI

MRI (*n* = 16) was taken at 14.3 ± 3.2 months (range, 12-24) post-harvest/injury on a 3T scanner (Skyra, Siemens, UK) using (1) a sagittal T1 spin echo sequence, (2) a sagittal proton density with fat saturation (PD-FS) sequence, (3) a coronal and axial PD-FS, and (4) a 3D sagittal PD-FS sequence.

MRIs were assessed by a consultant musculoskeletal radiologist with more than 20 years of experience in imaging cartilage repair. The exact location of the harvest site to assess was identified using the previously completed knee maps as a guide and the following features of the harvest site were scored: depth of repair fill (expressed as a percentage compared to the adjacent tissue), signal intensity (relative to adjacent native tissue, where 1 = isointense/normal, 2 = hyperintense, 3 = hypointense, and 4 = no cartilage present), and subchondral bone abnormalities (where 1 = normal, 2 = defect present, 3 = overgrowth/central osteophyte formation, 4 = bone marrow lesion, and 5 = subchondral cyst). In addition, another published MRI score (the mean total Area Measurement And DEpth & Underlying Structures score [AMADEUS^22^; score 0-100 where 100 is best]), designed for assessing cartilage defects, was also used to assess the harvest site.

### Arthroscopy and Biopsy

The repair tissue formed in the harvest site was assessed macroscopically during a follow-up arthroscopy at a mean of 15 ± 3.5 months (range, 13-25) post-harvest, using both the Oswestry Arthroscopy Score (O-AS, maximum score 10)^23^ and the International Cartilage Repair Society Arthroscopy Score (ICRS-AS, maximum score 12),^24^ where, for both scores, a higher score represents a better quality of repair (see **Supplementary Tables S1A** and **S1B** for a comparison of the different parameters scored within each system). A single core biopsy (1.8 mm diameter) of repair tissue formed at the site of the previously harvested donor cartilage was taken from each of the 16 patients during the same arthroscopic procedure using a juvenile bone marrow biopsy needle.

### Histology

Biopsies were snap-frozen in liquid nitrogen–cooled hexane and stored at −196°C until cryosectioning. Seven-micrometer-thick cryosections were collected onto poly-l-lysine-coated slides and stained with hematoxylin and eosin (H&E) or toluidine blue (TB) to assess general morphology and proteoglycan content of the repair tissue, respectively.^25^ Polarized light was used to assess collagen fiber organization and orientation. Sections were scored semi-quantitatively via both the Oswestry cartilage score (OsScore, a nominal score from 0 to 10 with 7 parameters)^26^ and the International Cartilage Repair Society (ICRS) II histological score (a visual analogue scale from 0 to 10 for each of the 14 parameters)^27^; for both systems, a higher score represents a better quality of repair tissue (see **Supplementary Table S2** for a comparison of the different parameters scored within each system).

### Immunohistochemistry

Cryosections were assessed for the presence and immunolocalization of collagen types I and II. In brief, cryosections were incubated with 4800 U/ml hyaluronidase (sheep testes, Sigma, Dorset, UK) for 2 hours prior to fixing in 4% formaldehyde for 10 minutes. Monoclonal antibodies against collagen type I (1:500, clone I-8H5, MP Biomedicals, Cambridge) and collagen type II (1:10, Developmental Studies Hybridoma Bank [DSHB] Cat# ciic1, RRID:AB_528164, IA, USA) were incubated for 60 minutes prior to the secondary biotinylated antibody (horse anti-mouse) for 30 minutes (Vectastain Elite ABC kit, Vector Laboratories, Peterborough, UK). Adjacent sections were incubated with a species-specific isotype-matched IgG as a negative control in place of the primary antibody. Non-specific binding and endogenous peroxidase activity were blocked using normal horse serum in phosphate-buffered saline (PBS) and 0.3% hydrogen peroxide in methanol, respectively. Sections were washed 3 times with PBS between steps and all steps were performed at room temperature. Labeling was enhanced with streptavidin-peroxidase (Vectastain Elite ABC kit, Vector Laboratories) and visualized with diaminobenzidine (DAB). Image analysis was performed on each section using the Colour Deconvolution and Threshold Plugins of the FIJI-ImageJ Software (Version 1.53), expressing the area of positive immunostaining as a percentage of the total area of the repair cartilage within the section.

### Statistical Analyses

Data were tested for normality using the Shapiro-Wilk normality test and subsequent analyses applied as appropriate. Parametric data were analyzed for statistical differences using a Student’s *t*-test. Non-parametric un-paired data, including categorical histological data (irrespective of normality), were analyzed for statistical differences using either a Mann-Whitney *U* test or a Kruskal-Wallis test (applying a Bonferroni’s *post hoc* correction). Correlations were analyzed using Spearman’s rank correlation, and categorical data were analyzed using Fisher’s exact test. Statistical analyses were performed using Analyse-it v4.50 (Analyse-it Software Ltd, Leeds, UK) and Prism 9.0.1 (GraphPad Software, San Diego, CA, USA). A 2-tailed *P*-value of less than 0.05 was considered statistically significant.

## Results

**Figure 1.**
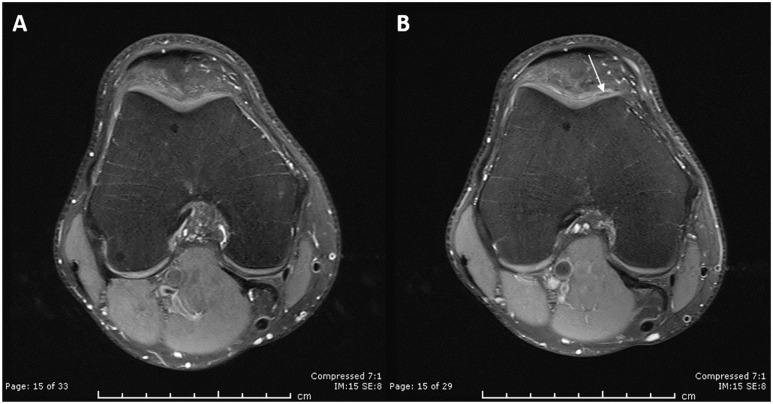
MRI analysis of a healed chondral harvest site. Representative protein density with fat saturation (PD-FS) MRI images taken from a single patient in the axial plane preoperatively (**A**) and 13-month post-injury (**B**). The harvest site on the craniomedial femoral trochlea was identified postoperatively using the knee maps created at the time of chondral harvest. A slight loss of signal in the cartilage (arrow) can be observed with normal bone marrow beneath.

There was no significant difference in age between male (38.2 ± 9.1 years, range = 22-51, **Table 1**) and female patients in this study (33.0 ± 10.4 years, range = 18-42, *P* = 0.356), but chondral harvests were significantly larger for males (299.8 ± 59.7 mg, range = 180-406) than females (218.7 ± 62.9 mg, range = 174-312, *P* = 0.039, **Table 1**). The mean depth of cartilage fill at the chondral injury site on MRI at 14 months post-harvest was 80.3% ± 26% (range = 25-100, **Table 2**). The overall signal intensity of the repair tissue was observed to be normal in 8 of 16 MRIs and hypointense in the remaining 8 of 16 MRIs. The underlying subchondral bone was normal in appearance in 11 of 16 MRIs and showed a small defect in 4 of 16 and a bone marrow lesion in 1 of 16. A subtle central osteophyte was also observed in one patient. No subchondral cysts were identified in this cohort of patients. Representative MRIs pre- and post-harvest can be seen in **Figure 1**. The mean AMADEUS score was 85 ± 15 (range, 45-100), equivalent to an AMADEUS Grade I (no defect).

**Table 2.** MRI Analysis of the Chondral Harvest Site 14 Months Post-Surgery.

Patient Number	Cartilage Fill (%)	Signal Intensity	Subchondral Bone
1	50	1	1
2	25	3	1
3	90	1	1
4	100	1	1
5	75	3	1
6	50	1	4
7	50	3	1
8	100	1	1
9	100	1	1
10	95	3	1
11	100	3	2
12	100	1	1
13	100	3	2
14	50	3	3
15	100	3	2
16	100	1	1

The chondral injury site was assessed on MRI for the following features: depth of repair fill (expressed as a percentage compared to the adjacent tissue), signal intensity (relative to adjacent native tissue, where 1 = isointense/normal, 2 = hyperintense, 3 = hypointense, and 4 = no cartilage present), and subchondral bone abnormalities (where 1 = normal, 2 = defect present, 3 = overgrowth/central osteophyte formation, 4 = bone marrow lesion, and 5 = subchondral cyst). One patient (patient 14) had a subtle central osteophyte.

**Figure 2.**
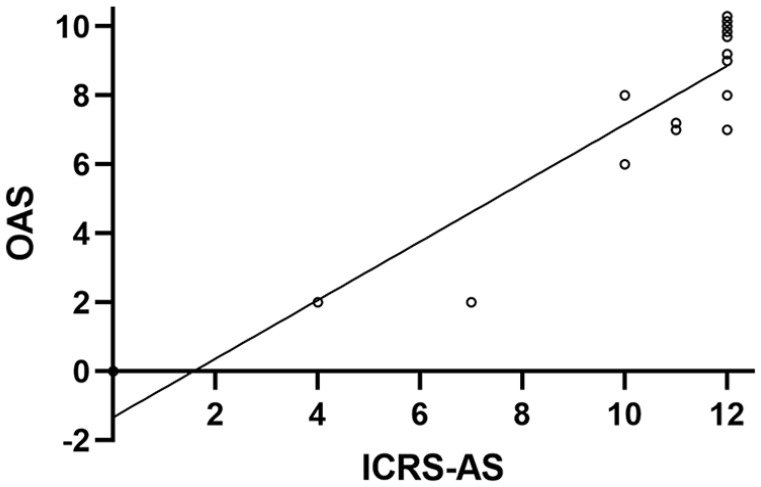
Comparing arthroscopy scores. There was a significant correlation between the Oswestry Arthroscopic Score (OAS) and the International Cartilage Repair Society Arthroscopy Score (ICRS-AS). A small degree of jitter in vertical direction has been added to visualize individual data points with identical coordinates.

Macroscopically, the mean O-AS and ICRS-AS of the repaired harvest sites were 7.2 ± 3.2 (range, 0-10) and 10.1 ± 3.5 (range, 0-12), respectively, being characterized as a Grade II quality of repair (**Supplementary Table S1A**),^24^ with no significant differences in either score between males and females. There was a significant correlation between the 2 arthroscopy scores (r = 0.92, *P* < 0.0001, **Fig. 2**). Lateral integration of the repair tissue as assessed arthroscopically was scored as complete in 12 of 16 and 11 of 16 patients by the O-AS and ICRS-AS, respectively. In 14 of 16 patients, the macroscopic surface of the repair tissue was scored as either smooth or having only fine fronds present and having a “pearly, hyaline-like or white appearance in colour.”^23^

**Figure 3.**
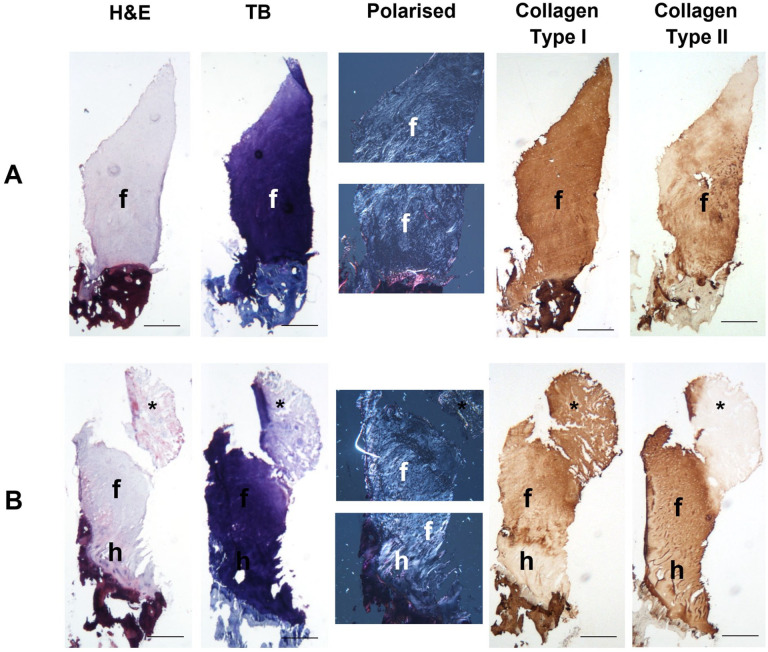
Histological and immunohistochemical analysis of the repair tissue formed post-harvest. Representative images of the repair tissue displaying tissue morphologies typical of solely fibrocartilage (**A**, Patient 3) and a mixture of hyaline and fibrocartilage (**B**, Patient 4). Cryosections were stained with hematoxylin and eosin (H&E) to assess general morphology and toluidine blue (TB) to assess glycosaminoglycan content. Collagen fiber orientation was assessed using polarized light and the localization of collagen types I and II was assessed with immunohistochemistry. Hyaline cartilage and fibrocartilage morphologies are depicted with the letters “h” and “f,” respectively and the asterisk (*) marks an area of fibrous tissue. Scale bars 500 μm.

While hyaline cartilage was predominant in 5 of 16 repair tissue biopsies, the microscopic morphology of the remaining 11 was variable (**Fig. 3**); based on collagen birefringence, a mixture of hyaline and fibrocartilage was found in 3 repair biopsies, predominantly fibrocartilage in 5 biopsies and fibrous tissue in 3 biopsies. No ectopic calcification was observed in any of the biopsies, although vascularization at varying degrees was observed in 8 of 16 biopsies (mean ICRS vascularization score = 7.7, range = 1.9-10, **Table 3**). Some cryosections were unfortunately lost during the immunostaining protocols and therefore analysis was only possible for 14 of 16 and 13 of 16 repair tissue biopsies for collagens type 1 and II, respectively. Collagen type I was detected in 12 of 14 biopsies (median percentage of the repair tissue being immunostained was 100, range = 10-100) and type II collagen in 10 of 13 biopsies (median percentage of area of repair tissue immunopositive was 100, range = 58-100, **Table 4**). Where staining for type II collagen was less than 100%, immunostaining occurred closest to the bone-cartilage interface.

**Table 3.** Histological Analyses of the Repair Tissue Formed 15 Months Post-Harvest.

	Mean Score ± SD (Range)	
(**A**) ICRS II parameter		
Tissue morphology	5.5 ± 3.1 (0.1-9.8)	
Matrix metachromasia	6.0 ± 3.6 (0.2-9.9)	
Cell morphology	4.3 ± 3.8 (0.1-9.4)	
Cell clusters	8.7 ± 2.0 (4.8-10)	
Surface architecture^a^	4.9 ± 3.3 (0.1-8.9)	
Basal integration^b^	8.2 ± 1.9 (3.7-10)	
Calcification front/tidemark^a^	5.6 ± 3.7 (0.4-10)	
Subchondral bone abnormalities^a^	7.6 ± 2.1 (1.3-9.2)	
Inflammation	10.0 ± 0 (10.0-10.0)	
Calcification	9.7 ± 1.0 (6.0-10.0)	
Vascularization	7.7 ± 3.3 (1.9-10.0)	
Surface/superficial assessment	4.1 ± 2.3 (0.6-8.5)	
Mid/Deep zone assessment	4.9 ± 2.6 (0.7-8.4)	
Overall assessment	4.2 ± 2.3 (0.4-8.4)	
		Number of Biopsies
(**B**) OsScore parameter		
Tissue morphology	Mostly hyaline	5
	Mix hyaline/fibrocartilage	3
	Mostly fibrocartilage	5
	Fibrous	3
Matrix metachromasia	Near normal	7
	Moderately normal	4
	Abnormal	5
Cell clusters	None	12
	<25% of cells	1
	>25% of cells	3
Surface architecture^a^	Near normal	3
	Moderately irregular	4
	Irregular	6
Basal integration^b^	Good	9
	Moderately irregular	3
	Poor	0
Calcification	Absent	14
	Present	2
Vascularization	Absent	8
	Present	8

Repair tissue biopsies (*n* = 16) were semi-quantitatively scored using (**A**) the International Cartilage Repair Society (ICRS) II histological score (a visual analogue scale from 0 to 10 for each of the 14 parameters, where a higher score indicates better-quality repair tissue)^27^ and (**B**) the Oswestry cartilage score (OsScore, a nominal score from 0 to 10 with 7 parameters).^26^ The ICRS II scores are displayed as mean ± standard deviation (SD) with the range and the OsScore is displayed as a nominal count per category. If an incomplete biopsy is obtained, some categories such as surface architecture, basal integration, tidemark, and subchondral bone abnormalities are unable to be scored, hence reduced *n*.

ICRS II = International Cartilage Repair Society II; SD = standard deviation.

a n = 13.

b n = 12.

**Table 4.** Immunolocalization of Collagen Types I and II in the Repair Tissue Formed 15 Months Post-Harvest.

Patient Number	% Area Immunostained	
	Collagen Type I	Collagen Type II
1	100	100
2	100	100
3	100	100
4	100	100
5	100	64
6	n/a	100
7	0	100
8	n/a	100
9	100	n/a
10	10	n/a
11	100	0
12	100	76
13	100	0
14	100	58
15	20	n/a
16	100	0

Repair tissue biopsies (*n* = 16) were examined for the presence and localization of collagen types I and II via immunohistochemistry. Image analysis was performed on each section to express the area of positive immunostaining as a percentage of the total area of the repair cartilage within the section.

n/a = loss of cryosection during immunostaining.

The mean “overall parameter” (indicating how closely the repair tissue resembles normal articular cartilage) for the ICRS II histology score was 4.2 ± 2.3 (range, 0.4-8.4) and the mean OsScore was 6.1 ± 2 (range, 2.5-9.4), with no significant differences between the sexes. The presence of any hyaline cartilage within the repair tissue was associated with a significantly higher ICRS-AS (median 11) than if no hyaline cartilage was present (median = 7.5, *P* = 0.04); there was a similar trend with the O-AS, but this was not significant (median = 9.5 vs. 7.5 for with and without hyaline cartilage, respectively). The total OsScore correlated significantly with both the O-AS and ICRS-AS (r = 0.49 and 0.52; *P* = 0.05 and *P* = 0.04, respectively), but the overall parameter for the ICRS II histology score did not. No other histological parameters were found to correlate with either arthroscopic score.

A higher proportion of repair tissue with a hypointense signal on MRI (6/8 biopsies) exhibited hyaline cartilage (solely or together with some fibrocartilage) compared with only 2 of 8 biopsies where the MRI signal was normal, but this did not reach significance (RR = 3.0, 95% CI = 0.85 to 10, *P* = 0.132). Although biopsies taken from hypointense regions also demonstrated a higher mean overall ICRS II and OsScore histology score (5.3 ± 1.9 and 7.1 ± 1.6, respectively) than those with a normal signal intensity (3.3 ± 2.1 and 5.2 ± 1.9, respectively), this was not quite significant (*P* = 0.085 and *P* = 0.052, respectively). In addition, there was no significant difference in matrix metachromasia between biopsies taken from hypointense regions compared with those from normal intensity regions (6.6 ± 3.1 and 5.4 ± 4.0, respectively, *P* = 0.506 for ICRS II; *P* = 0.590 for OsScore). Finally, bony changes observed on MRI did not significantly affect the quality of the repair tissue formed; there was no significant difference in either the overall ICRS II or OsScore histology score for repair tissue obtained from harvest sites with normal subchondral bone (*n* = 11; 4.4 ± 2.1 and 5.9 ± 1.8, respectively) compared with those with a bony change (*n* = 5; 3.8 ± 2.9 and 6.3 ± 2.6, respectively, *P* = 0.721 for ICRS II; *P* = 0.730 for OsScore).

## Discussion

For almost 30 years, orthopedic surgeons have been performing ACI in patients for chondral knee defects with the belief that the chondral harvest “does no harm.” Anecdotal evidence of course supports this, but the ability to study and assess the chondral harvest as an individual “site” is challenging due to the comorbidities of other (treated) defects in the joint. Here, we present evidence of a good to excellent level of repair in these harvest sites, both radiographically and histologically. The belief that articular cartilage has a poor inherent ability for repair has been supported by observations in animals where a controlled chondral injury in large animal studies has mostly demonstrated a very poor natural healing response,^28,29^ in contrast to a much better healing response following an osteochondral injury, where there is penetration into the underlying bone.^16,30-32^ In both chondral injuries and those extending into the subchondral bone, location has been shown to have an impact on the healing response, with osteochondral defects in large animal models showing significantly better natural repair in the trochlea compared with the medial femoral condyle in the knee joint.^30,31^ In keeping with this, the present study clearly demonstrates that injured articular cartilage in human trochlea can also heal naturally, as evidenced both macroscopically and microscopically.

The size of the “defects” created following the chondral harvest that we studied was not small. Although not measured at the time of surgery, it is estimated that the chondral harvest sites would have had an original surface area equivalent to a circular defect with a diameter of 10 to 15 mm, calculated from the recorded weight of the harvested tissue, the size of instrument used, and published protocols.^33^ These are of a comparable size to symptomatic defects in other locations of the joint that would otherwise be treated, for example, with bone marrow stimulation techniques.^34^ Pre-clinical models used for investigating chondral/osteochondral repair have identified a “critical defect size” for each model system, whereby endogenous repair capacity fails and degeneration is likely, but this appears to not exist in humans, at least in the location studied here. The articular cartilage in the stifle joint of a horse is suggested to be the most synonymous with human articular cartilage and is estimated to have a 9-mm critical size defect in any location in the stifle.^15,35^ The nature of harvesting cartilage as a source of cells in ACI is to create an injury small enough to remain asymptomatic; our previous work where cartilage was sourced from asymptomatic knees to treat other joints supports this with no significant change in Lysholm score up to 4.8 years post-harvest.^18^ To our knowledge, the present study is the first time the structural quality of the repair tissue formed at such a site has been assessed systematically. We demonstrate a good structural outcome of endogenous repair following chondral harvests in the peripheral trochlea which are of a comparable size to symptomatic defects in other locations. This is not to say, however, that all defects of this size or indeed in this location will repair to the same capacity.

It is believed that osteochondral injuries have a greater potential for natural repair than simple chondral injuries due to the breaching of the subchondral bone in the former, which allows an influx of bone marrow stromal cells that could contribute to the repair process. During the harvesting procedure for ACI, every effort is made not to rupture the calcified cartilage and/or underlying subchondral bone, although this cannot be guaranteed. In the current study, we did not find any association between the overall quality of the natural repair (when viewed arthroscopically) and the integrity of the tidemark (as assessed histologically), nor were any subchondral cysts identified on MRI in any of the subjects investigated. Our study demonstrated a normal, healthy appearance of the subchondral bone on MRI in 69% of the patients with no apparent inflammatory bone marrow signal change beneath the injury site. Large animal studies assessing chondral injuries have demonstrated changes in the underlying subchondral bone up to 18 months injury.^12,14^ Although we observed post-harvest bony changes on MRI in 5 of 16 patients, this does not necessarily indicate that the subchondral bone was breached during the procedure and cannot therefore be assumed to contribute to the mechanism of repair in those patients.

The source of cells which might elicit a repair response is unclear. While cells from the bone marrow are likely to be involved with repair of an osteochondral defect, there are other cell sources within the joint which may enable repair. For example, the synovium is a specialized connective tissue, lining the inside of a synovial joint such as the knee. It is a rich source of different cell types and is very reactive in conditions such as OA and rheumatoid arthritis. Cells from the synovium have previously been shown to invade areas of damaged cartilage and are hypothesized to assist in the repair process,^9,36-38^ with mesenchymal stromal cells (MSCs) derived from the synovium having been shown to have a superior ability for chondrogenesis compared with bone marrow–derived MSCs.^39^ Synovial infiltrates could also explain the high incidence of vascularization observed within the naturally occurring repair tissue, and these may disappear with time as the tissue matures and remodels, perhaps in response to load bearing.^40^

Here, we have demonstrated the production of both collagen types I and II in the naturally repaired tissue, indicating a cartilaginous matrix has been produced, albeit with a higher degree of type I collagen than is seen in normal adult articular cartilage.^41^ This is a trait also seen in patients who have had ACI to treat condylar cartilage defects, with repair tissue containing considerable amounts of type I collagen, apparently maturing with time to contain greater amounts of type II collagen later post-treatment, as assessed both immunohistochemically^42^ and biochemically.^43^

The chondral harvest site in this study repairs with a cartilaginous tissue resembling that of healthy, native hyaline cartilage in the majority of patients when observed both radiographically and arthroscopically, similar to that generated following repair such as ACI.^9,42^ On MRI, the injury site exhibited a normal intensity signal, similar to adjacent healthy cartilage, in half the patients assessed, indicating the repair cartilage to be of a similar quality and structural makeup. Previously, we reported that neither the structure of repair tissue (on MRI) nor the overall signal intensity had any correlation with microscopic tissue morphology.^9^ The apparent association therefore in the current study between a hypointense signal on MRI and a better-quality tissue morphology is surprising, if one assumes that a signal intensity relates simply to water content (which is associated with proteoglycan content). However, MR signal can also be influenced by the extracellular matrix organization, in addition to absolute differences in water or proteoglycans.^44^ Certainly, there was no notable difference in proteoglycan content as observed metachromatically in biopsies obtained from the joints with different MRI intensities for the repair tissue. In addition, as also seen with repair tissue following procedures such as ACI, the repair tissue as assessed via its microscopic morphology was variable between/within the samples, highlighting a similar level of unpredictability for the quality of natural repair achieved. Of note, perhaps, was the fact that a seemingly higher percentage of biopsies comprised poor-quality repair tissue with fibrous morphology (3/16; 19%) and extensive vascularization (8/16; 50%) in these naturally repaired sites compared to between 0% and 5% in studies of ACI repair tissue.^9,26^

As has been found in ACI-treated cartilage defects, the current study demonstrates good lateral integration of the naturally repaired tissue with the surrounding native cartilage.^9^ It has been shown that following either a chondral or an osteochondral injury, chondrocytes in the (healthy) adjacent native cartilage may contribute to the repair of the defect, starting from the top edges of the defect,^16,28^ possibly via the activation of progenitor cells which are known to reside in articular cartilage, particularly in the surface zone.^45,46^ Chondrocytes within cartilage can respond to an insult and injury via a series of changes in gene expression and activation of signaling factors such as bone morphogenetic proteins (BMPs), Wnt-signaling proteins, and signaling proteoglycans such as agrin.^47-49^ Agrin has been shown to support cartilage regeneration in both small and large animal models by the induction of chondrogenic differentiation in synovial MSCs via modulation of Wnt signaling.^49,50^

In conclusion, the present study provides clear evidence for human articular cartilage to produce good-quality repair tissue in response to a chondral harvest. This is in keeping with previous studies whereby a chondral harvest during ACI was not considered to be associated with significant joint morbidity.^18^ Our observation of this natural repair response was restricted to the trochlea, a location less commonly affected by early OA in humans, and cartilage at other locations in the joint may therefore have a different ability to repair spontaneously post-injury. However, understanding more about the mechanisms and factors influencing such natural healing and the possible effects that joint loading or instability may have on the process could provide useful information for guiding improved treatments for cartilage defects in the future.

## Supplemental Material

sj-docx-1-car-10.1177_19476035221149523 – Supplemental material for Histological and Radiological Assessment of Endogenously Generated Repair Tissue In Vivo Following a Chondral Harvest Click here for additional data file. Supplemental material, sj-docx-1-car-10.1177_19476035221149523 for Histological and Radiological Assessment of Endogenously Generated Repair Tissue In Vivo Following a Chondral Harvest by Helen S. McCarthy, Bernhard Tins, Peter D. Gallacher, Paul Jermin, James B. Richardson, Jan Herman Kuiper and Sally Roberts in CARTILAGE
