# Supplementary material for: Histological and Radiological Assessment of Endogenously Generated Repair Tissue In Vivo Following a Chondral Harvest
Source: Cartilage. 2023 Jan 26;14(1):48–58. doi: 10.1177/19476035221149523 (PMC10076898; doi:10.1177/19476035221149523)
Supplement: sj-docx-1-car-10.1177_19476035221149523 – Supplemental material for Histological and Radiological Assessment of Endogenously Generated Repair Tissue In Vivo Following a Chondral Harvest [file sj-docx-1-car-10.1177_19476035221149523.docx]

**Supplementary Material (*if any*)**

**Supplementary Table S1. Comparison of the parameters assessed in the ICRS and Oswestry arthroscopy scores.**

The International Cartilage Repair Society (ICRS-AS; Table S1A) and Oswestry (OAS; Table S1B) arthroscopy scores both macroscopically assess different parameters of cartilage repair, with both systems resulting in a single, totalled score where a higher score represents better quality repair, more akin to native hyaline cartilage ^23,24^. The ICRS-AS also provides an “overall repair assessment”, depending on the totalled score of the parameters assessed.

**Table S1A**

| ICRS-AS Parameter | | Score |
| --- | --- | --- |
| Degree of defect repair | Level with surrounding cartilage | 4 |
|  | 75% repair of defect depth | 3 |
|  | 50% repair of defect depth | 2 |
|  | 25% repair of defect depth | 1 |
|  | 0% repair of defect depth | 0 |
| Lateral integration | Complete integration with surrounding cartilage | 4 |
|  | Demarcating border < 1 mm | 3 |
|  | 3/4th of graft integrated, 1/4th with a notable border > 1 mm width | 2 |
|  | 1/2 of graft integrated with surrounding cartilage, 1/2 with a notable border > 1 mm | 1 |
|  | From no contact to 1/4th of graft integrated with surrounding cartilage | 0 |
| Macroscopic appearance of surface | Intact smooth surface | 4 |
|  | Fibrillated surface | 3 |
|  | Small, scattered fissures or cracks | 2 |
|  | Several small or few large fissures | 1 |
|  | Total degeneration of grafted area | 0 |
| Overall repair assessment (score total) | Grade I: normal | 12 |
|  | Grade II: nearly normal | 11–8 |
|  | Grade III: abnormal | 7–4 |
|  | Grade IV: severely abnormal | 3–1 |

**Table S1B**

| OAS Parameter | | Score |
| --- | --- | --- |
| Degree of defect fill | Level with surrounding cartilage | 2 |
|  | Raised | 1 |
|  | Below | 0 |
| Lateral integration | Complete | 2 |
|  | Minor disruption (<25% of area) | 1 |
|  | Major disruption (>25% of area) | 0 |
| Macroscopic appearance of surface | Smooth | 2 |
|  | Fine fronds | 1 |
|  | Severe fronds/fibrillation | 0 |
| Colour of graft | Pearly, hyaline-like | 2 |
|  | White | 1 |
|  | Yellow bone | 0 |
| Stiffness on probing | Normal compared to adjacent cartilage | 2 |
|  | Softer | 1 |
|  | Very soft/hard | 0 |
| Score total |  | 0-10 |

**Supplementary Table S2. Comparison of the parameters assessed in the ICRS II and OsScore histology scoring systems.** The ICRS II histology score assesses 13 different parameters, with a 14^th^ parameter assessing the scorer’s “overall” opinion on the quality of the sample being examined ^27^. Each parameter is scored on a visual analogue scale (from 0-10). The OsScore is a categorical scoring system, assessing 7 different parameters, whose score is totalled (max 10; score parameters are weighted as follows: Tissue morphology (Mostly hyaline=3, hyaline/fibrocartilage mix=2, mostly fibrocartilage=1, fibrous tissue=0); Matrix metachromasia (near normal=1, moderately normal = 0.5, abnormal=0); Clusters (none=1, <25% of cells=0.5, >25% of cells=0); Surface architecture (near normal=2, moderately irregular=1, very irregular=0); Basal integration (good=1, moderately irregular = 0.5, poor=0); Calcification (absent=1, present=0); Vascularisation (absent=1, present=0) ^26^. For both systems, a higher score represents a more “normal”, better quality repair tissue.

| Histology Scoring Parameter | ICRS II | OsScore |
| --- | --- | --- |
| Tissue Morphology | X | X |
| Matrix Metachromasia | X | X |
| Cell Morphology | X |  |
| Cell Clusters | X | X |
| Surface Architecture | X | X |
| Basal Integration | X | X |
| Calcification Front / Tidemark | X |  |
| Subchondral Bone Abnormalities | X |  |
| Inflammation | X |  |
| Calcification | X | X |
| Vascularisation | X | X |
| Surface / Superficial Assessment | X |  |
| Mid / Deep Zone Assessment | X |  |
| Overall Assessment | X |  |
